# Do More with Less? Lobectomy vs. Segmentectomy for Patients with Congenital Pulmonary Malformations

**DOI:** 10.3390/jcm12165237

**Published:** 2023-08-11

**Authors:** Beatrice Trabalza Marinucci, Cecilia Menna, Paolo Scanagatta, Silvia Fiorelli, Matteo Tiracorrendo, Giuseppe Naldi, Alessandro Inserra, Francesco Macchini, Erino Angelo Rendina, Mohsen Ibrahim

**Affiliations:** 1 Thoracic Surgery Sant’Andrea Hospital, La Sapienza University, 00186 Rome, Italy; cecilia.menna@uniroma1.it (C.M.); tiracorrendomatteo@gmail.com (M.T.); erinoangelo.rendina@uniroma1.it (E.A.R.); mohsen.ibrahim@uniroma1.it (M.I.); 2 Thoracic Surgery—Morelli Hospital, ASST Valtellina e Alto Lario, 23100 Sondalo, Italy; paoscan@hotmail.com (P.S.); giuseppe.naldi@asst-val.it (G.N.); 3 Anesthesiology and Intensive Care, Sant’Andrea Hospital, La Sapienza University, 00186 Rome, Italy; silvia.fiorelli@uniroma1.it; 4 General and Thoracic Surgery—Bambino Gesù Children’s Research Hospital IRCCS, 00165 Rome, Italy; alessandro.inserra@opbg.net; 5 Paediatric Surgery—Niguarda Hospital, ASST Grande Ospedale Niguarda, 20162 Milan, Italy; francesco.macchini@ospedaleniguarda.it

**Keywords:** Congenital Pulmonary Malformations, thoracic surgery, lobectomy, pulmonary sequestration, congenital cystic adenomatoid malformation

## Abstract

Background: Congenital Pulmonary Malformations (CPMs) are rare benign lesions potentially causing infective complications and/or malignant transformation, requiring surgery even when asymptomatic. CPMs are rare in adulthood but potentially detected at any age. There is not a consensus on the correct extent of resection in both adults and paediatrics. This retrospective multicentric study aims to identify the appropriate surgical resection to prevent the recurrence of the related respiratory symptoms. Methods: Between 2010 and 2020, a total of 96 patients (adults and pediatrics) underwent surgery for CPMs in 4 centers. A 2:1 propensity score matching (considering sex and lesion side) was performed, identifying 2 groups: 50 patients underwent lobectomy (group A) and 25 sub-lobar resections (group B). Clinical and histopathological characteristics, early and late complications, and symptom recurrence were retrospectively analyzed and compared between the two groups by univariate and multivariate analysis. Results: Patients who underwent lobectomy had a statistically significant lower rate of recurrence (4% vs. 24% of group B, *p* = 0.014) and a lower rate of intraoperative complications (*p* = 0.014). Logistic regression identified sub-lobar resection (*p* = 0.040), intra- and post-operative complications (*p* = 0.105 and 0.022),and associated developed neoplasm (*p* = 0.062) as possible risk factors for symptom recurrence after surgery. Conclusions: Pulmonary lobectomy seems to be the most effective surgical treatment for CPMs, guaranteeing the stable remission of symptoms and a lower rate of intra- and postoperative complications. To our knowledge, this is one of the largest studies comparing lobectomy and sub-lobar resections in patients affected by CPMs, considering the low incidence worldwide.

## 1. Introduction

CPMs (Congenital Pulmonary Malformations) are a wide spectrum of congenital lung benign lesions such as pulmonary sequestration (PS), congenital cystic adenomatoid malformation (CCAM), congenital lobar emphysema, bronchial atresia, and others intermediary forms. 

CCAMs are the most frequent, occurring in 25% of all CPMs [1] and consisting of hamartomatous cystic replacement of the normal lung parenchyma. CCAMs are gland-like cystic lesions of different sizes first classified by Stocker et al. [2] as type 1 (large cysts of 3–10 cm in diameter), type 2 (smaller cysts of <2 cm), and type 3 (minute cysts of <0.3 cm). Later, two other types were added: type 0 (solid malformation often not compatible with life) and type 4 (few peripheral cysts lined by alveolar epithelium). 

**Figure 1:**
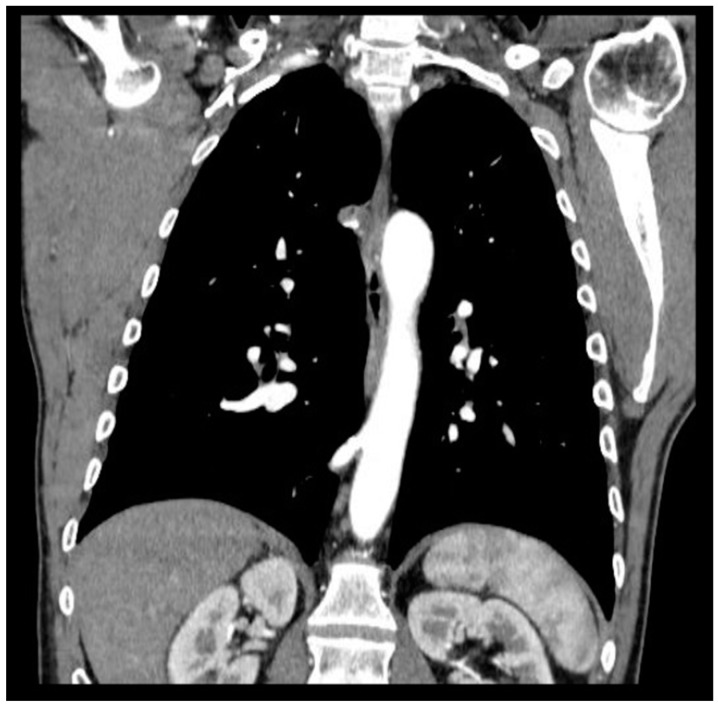
CT evidence of systemic arterial vascularization from descending thoracic aorta.

Pulmonary sequestration (PS) represents 0.15–6.45% of CPMs [3]; it is characterized by a mass of non-functioning lung tissue separated from the normal tracheobronchial tree and receiving vascular supply from a systemic artery (Figure 1). It is classified as intra-lobar PS (IL-PS) when it is incorporated in the parenchyma of a lobe and as extra-lobar PS (EL-PS) when it is separated from the adjacent normal parenchyma by its own pleural envelope.

CPMs represent anomalies occurring because of the abnormal embryonal development of the bronchial tree and they can often be associated with other congenital anomalies or with each other. In fact, CCAM is described in 50% of extra-lobar PS and in 15% of intra-lobar PS [4].

In the past decades, the spread of prenatal ultrasonography has led to a progressive increase in the antenatal detection of CPMs, confirmed by radiological images (chest Rx or CT scan) early after birth.

Most neonates are asymptomatic at birth (>75%). The clinical presentation and severity depend on the extent and on the localization of the lesion [5,6]. CPMs clinically present with respiratory symptoms (such pneumonia, hemoptysis, productive coughing, recurrent wheeze) due to infective complications, occurring most frequently at an average age of 7 months or later during growth. In fact, prenatal diagnosis is sometimes missed, and detection may occur later, either by chance or because of unexplained recurrent or persistent respiratory symptoms or signs, occurring beyond infancy, in adolescence, and/or adulthood. As previously reported in the literature, the most frequent symptoms related to CPMs are pneumonia, recurrent bronchitis, bronchiolitis, severe cough, hemoptysis, dyspnea, and respiratory distress [7,8]. 

Surgical treatment is mandatory for symptomatic lesions. On the contrary, the management of asymptomatic lesions is rather controversial, as some authors sustain a conservative approach before respiratory symptoms occur. Nevertheless, there is wide concern in literature about the indication of surgical resection even for asymptomatic lesions, to prevent recurrent respiratory symptoms, to ensure a safer surgery before inflammation complicates the anatomy of the lesion and, finally, to avoid the rare but possible malignant transformation [6]. A preliminary study from Rotterdam evaluating pulmonary function in children undergoing pulmonary lobectomy before and after the age of 2 years, showed no differences. Early resection before the development of respiratory complications may also facilitate the thoracoscopic approach of surgical treatment in experienced hands. 

Consequently, surgery is indicated early after lesion detection. Moreover, considering that detection is most frequent in neonates and infants, parenchyma-sparing techniques have been proposed depending on the lesion’s size and localization. However, the potential lung growth in paediatric patients is described to justify an early lobectomy [7]. To date, there is not a consensus indicating the correct extent of surgical resection between lobectomy and sub-lobar resection for CPMs in adult or paediatric patients. 

This retrospective multicentric study aims to identify the appropriate surgical treatment to prevent the recurrence of respiratory symptoms related to the lesions.

## 2. Materials and Methods

This multi-institutional retrospective observational study included 96 consecutive paediatric and adult patients surgically treated between 2010 and 2020 for CPMs in 4 centres: La Sapienza University of Rome, ASST Valtellina e Alto Lario of Sondrio, Bambino Gesu ‘Children’s Research Hospital of Rome, and IRCCS Ca’ Granda Foundation—Policlinico of Milan. 

Lesions were detected radiologically by prenatal ultrasonography of the second pregnancy trimester, then confirmed by computed tomography (CT) scan within the first 3 months after birth, or immediately in symptomatic lesions. Lesions detected prenatally were surgically treated within the first year of age, as previously reported [8]. In all the other cases, lesions were detected incidentally or when symptomatic by CT scan and treated early after the diagnosis.

Preoperative assessment included respiratory functional tests (spirometry and blood gas analysis) and cardiovascular tests (electrocardiography and echocardiography when required). In infants under 5 years old, infant pulmonary function test was used to assess functional capacity [9].

To minimize selection bias, 2:1 propensity score matching was performed based on predetermined confounders and baseline characteristics (sex and lesion side) to identify two homogenous groups of patients: finally, 75 patients with homogenous characteristics were selected and divided into group A (50) treated by lobectomy; group B (25) treated by sub-lobar resections (segmentectomies). Based on the propensity score matching, 21 patients were excluded because they did not match the variables. Videothoracoscopy was performed with a bi-portal access. Thoracotomy was performed with a muscle-sparing technique when VATS was not eligible because of lesion dimensions, localization, and surgical expertise to manage intra-operative complications. The choice to perform lobectomy or sub-lobar resection was made considering radiological and intra-operative characteristics of the lesion (lesions distant < 1 cm from the fissure and/or bigger than 4 cm of maximum diameter at pre-operative CT scan and/or involving two or more pulmonary segments, were considered for lobectomy).

ICU (Intensive Care Unit) admission depended on patient characteristics, comorbidities, intra-operative events, and complications; so surgical team (surgeons and anaesthesiologists) make a decision on its necessity case by case. 

An accurate histopathological analysis was conducted. Paediatric patients underwent clinical follow-up once a year up to 18 years and then addressed their general practitioner, who indicated new surgical consulting in case of relapse. Adult patients were followed up for the first year; then, the general practitioner was addressed as well. Demographics characteristics, diagnosis technique, histology, time of surgery, length of hospital stay, length of chest tube permanence, intra-operative and post-operative complications, and symptom recurrence were compared between the two groups. 

The study was approved by the institutional review board of Sant’Andrea Hospital (PROT. N. 188 SA/2022) and informed consent was obtained before surgery. 

Data were prospectively collected and stored in Excel database (Microsoft Corp, Redmond, WA, USA). Quantitative variables were expressed as mean ± standard deviation and compared using *t*-test. Nominal variables were expressed binarily (presence—1 or absence—0) and compared by Chi square, after Fisher’s exact test was performed. *p*-values less than 0.05 were considered statistically significant.

A univariate logistic regression analysis was performed to derive crude estimates of association between predictors and outcomes. After univariate analysis, variables with *p*-values less than 0.05 were included in a multivariate logistic regression model to identify potential independent protective or risk factors for symptom recurrence. The adjusted odd ratios (ORs) and 95% confidence intervals (CI) were calculated to estimate and measure the association using 1000 bootstrapping samples.

Data were analyzed using statistical package SPSS, version 25.0 (SPSS Software, IBM Corp., Armonk, NY, USA).

## 3. Results

The general characteristics of the patients and postoperative results are shown in Table 1.

The average age was 12.7 years in group A and 4.2 years in group B, with a statistically significant difference in the *t*-test analysis (*p* = 0.012). 

There were 30 (60%) male patients in group A and 12 (48%) in group B. 

In group A, 42 (84%) patients had a prenatal diagnosis and 17 (34%) were symptomatic at the diagnosis, while in group B, 18 (72%) patients had a prenatal diagnosis and 6 (24%) were symptomatic. 

The lesion was right-sided in 28 (56%) patients in group A and in 10 (40%) patients in group B. 

The mean operatory time was 167 min in group A and 161 min in group B, without a statistical significance in the *t*-test analysis.

VATS was performed in 31 (62%) patients in group A and in 16 (64%) in group B, all in the last 10 years. Mini-thoracotomy was performed in 19 (38%) patients in group A and in 9 (36%) in group B.

Histopathological analysis detected 29 (58%) cases of combined lesions (PS and CCAM) in group A and 8 (32%) cases in group B, with a significant statistical difference (*p* = 0.030). Equally, CCAM alone was detected in 19 (38%) of patients in group A and in 2 (8%) in group B, with a significant statistical difference (*p* = 0.005).

Intra-operative complications (pulmonary laceration, hematoma, hemorrhage) were significantly higher in sub-lobar resection group: 6 (24%) in group B vs. 2 (4%) in group A, (*p* = 0.014).

Post-operative complications (persistent air leak, slow re-expansion, bleeding, chest wall hematoma, prolonged liquid leaks) occurred in 5 (10%) of patients in group A vs. 3 (12%) in group B, without a significative difference. 

Hospital stay was <7 days for 38 (76%) in group A and for 16 (64%) in group B, without a significant difference. The average hospital stay was 8 days for group B (median of 7 days) and 8.80 days for group A (median of 5 days), without a significant difference in *t*-test analysis. 

The length of chest tube permanence was >7 days for 6 (12%) patients in group A and for 9 (36%) patients in group B, with significant difference (*p* = 0.018). An average of 3.7 days for group A (median of 3 days) and 6.6 days for group B (median of 6 days), with a significant difference in t-test analysis (*p* = 0.005). 

Finally, the recurrence of respiratory symptoms related to the lesion was higher in the sub-lobar group (6; 24% in group B vs. 2; 4% in group A; *p* = 0.002) as it is shown in Table 2, which reports differences among patients who experienced symptom recurrence and patients who did not. 

The average follow-up period was 10.5 years.

Univariate analysis identified the following risk factors for symptom recurrence after surgical treatment: pulmonary sequestration (*p* = 0.002), sub-lobar resection (*p* = 0.019), intra-operative (*p* = 0.020) and post-operative (*p* = 0.020) complications, and associated neoplasm (*p* = 0.017). Multivariate logistic regression confirmed the following risk factors for symptoms recurrence after surgical treatment: sub-lobar resection (*p* = 0.040), post-operative complications (*p* = 0.022), and associated developed neoplasm (at the edge of statistical significance) (Table 3).

## 4. Discussion

CPMs are rare benign lesions involving 30–42 cases per 100.00 inhabitants per year [9,10,11] with an increased risk of infective complications. In the last decades, the extensive use of prenatal ultrasonography has led to a progressive increase in the antenatal detection of CPMs. For this reason, the highest incidence of the pathology occurs in pediatric patients, but the diagnosis may be missed until later in life, even in adulthood. Surgery is mandatory for symptomatic lesions to solve respiratory symptoms, to prevent infective complications, and to avoid the rare risk of malignant transformation. Nevertheless, there is a wide concern in the literature on surgical indication, even for asymptomatic lesions, to prevent late infections and/or malignancies, to guarantee more time for alveolar compensatory growth, and to reduce the risk for emergency surgery. The incidence of post-operative complications is assumed to be lower after early elective operation for CPMs than after an urgent intervention for CPMs when infective complications occurred [10]. 

Surgery for CPMs is indicated early after lesion detection and many studies in literature discuss surgical treatment options (thoracotomy vs. VATS) [12,13,14,15]; however, there is no standardized consensus on the extension of surgical resection between lobectomy and sub-lobar resections. A study by Baird et al. [12] recommends lobectomy (recommendation: weakly agree) as the procedure of choice, especially when the lesion is confined to a single lobe. There are no guidelines in the literature on the correct extension of surgical resection but considering the best practice, formal lobectomy is superior to segmentectomy. The present study investigates the possibility of identifying a consensus among surgeons who approach these pathologies. 

In our series, the patients who underwent sub-lobar resections had an average age of 4.2 years vs. 12.7 years in the lobectomy group. Theoretically, it is possible to speculate that parenchyma-saving resection is the optimal choice for children for preserving total lung capacity. Nevertheless, it should be noted that parenchyma-saving resection can be complicated by prolonged air leak in the early postoperative period; moreover, in patients with residual lesion, malignant transformation as well as recurrent infections could develop during the follow-up period, even requiring the surgery to be repeated. However, it is demonstrated that neonates and infant patients have the potential for lung growth within the first years of life [16]. Infants and children tolerate lobectomy well, so that the total lung volume and gas exchange capacity return toward normal during somatic maturation [7]. For these reasons, the ideal extent of resection is still debated [17].

**Figure 2:**
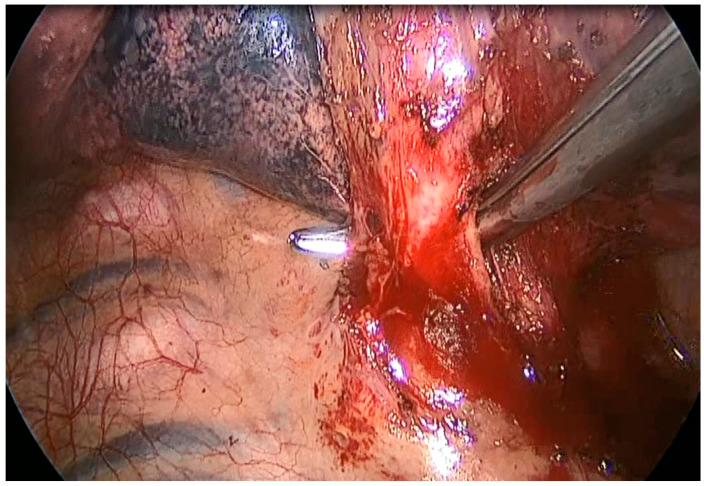
Intraoperative isolation of an extralobar pulmonary sequestration’s arterial branch derived from thoracic aorta.

Both video-thoracoscopy (VATS) and thoracotomy were described for the treatment of CPMs. VATS is the procedure of choice; it is demonstrated to be safe and effective, and it is recommended to reduce morbidity related to thoracotomy, guaranteeing cosmetic benefit, and reducing pain [18]. Thoracotomy allows easy access to hilar structures using different intercostal spaces, making the management of anomalous vascularization and the variable size of the lesions easier than VATS. Moreover, dense adhesions due to recurrent infections of the lesion can make VATS dissection more difficult and thoracotomy is sometimes required to better manage an eventual vessel injury when the aberrant or varicose bronchial arteries are not well defined by preoperative imaging (Figure 2); therefore, it is mandatory to consider conversion whenever necessary. In our series, VATS was performed in 31 (62%) patients in group A and 16 (64%) patients in group B in the last 10 years, reflecting the increasing surgical expertise in mini-invasive surgery over the years. Conversion was required only for two patients to manage intra-operative complications (anomalous vessel laceration; tough adhesions). No differences in outcome were found in comparison with the open access approach in the present series.

The results reported are in line with previous smaller published studies (Kim et al., Stanton et al.) [16,17] reporting the superiority of lobectomy for the stable remission of symptom recurrence.

Our comparative analysis showed that symptom recurrence after surgical treatment for CPMs was lower in the lobectomy group compared to the sub-lobar group, with statistical significative difference (*p* = 0.014). Even intra-operative complications (pulmonary laceration, hematoma, hemorrhage) were more frequent in sub-lobar resections (24% vs. 4%, *p* = 0.014), probably because these kinds of lesions rarely are limited to an anatomic pulmonary segment. This result probably explains the longer period of chest tube permanence in group B than in group A, with a statistically significant difference. There was not a significant difference in post-operative complications, surgical time, or hospital stay in the two groups, although there was a difference in chest tube drainage permanence, which was longer in the sub-lobar group, probably due to the more frequent intra-operative complications registered in the abovementioned group; nevertheless, the prognosis of post-operative complications was good and patients that experienced post-operative complications had a good recovery at the mean follow-up time. 

The comparison of histopathological samples showed an increased frequency of CCAM (isolated lesion or associated with sequestration) in the lobectomy group than in the sub-lobar resection group. Histopathological analysis represents the definitive diagnosis [19] because radiological definition is not always conclusive. In fact, contrast chest CT is considered the most accurate examination, but hyperlucent lesions presuppose an overlapping spectrum and consensus and cooperation between radiologists and histopathologists are of paramount importance for the definitive diagnosis [20]. Therefore, prenatal imaging is not predictive of post-natal histology and surgical resection is also recommended to obtain a final diagnosis of the lesion, to distinguish isolated lesions from associated CPMs, generally related to a higher risk of infective complications and/or malignant transformation [21].

Analyzing the causes of symptom recurrence (present in 8 patients, 11% of the entire population), the presence of isolated sequestration (*p* = 0.062), intra- and post-operative complications (*p* = 0.035), associated neoplasm at histopathological analysis (*p* = 0.014), and sub-lobar resections (*p* = 0.014) seemed significantly related to the recurrence. 

These variables were then analyzed in a univariate analysis that confirmed them as potential risk factors for symptom recurrence. The multivariate logistic regression confirmed the variables shown in Table 3 as risk factors for symptom recurrence. 

The recurrence of symptoms was defined by the occurrence of productive cough, fever, hemoptysis related to pneumonia, bronchiolitis, and/or recurrent bronchitis during the follow-up period [8]. According to Calzolari et al. [10]., symptoms such asthma, recurrent wheeze, and/or respiratory distress presenting after surgery are not considered a recurrence of symptoms caused by the lesions, but symptoms related to the same congenital developmental abnormality that causes the formation of CPMs. Patients presenting with these latter symptoms were not considered affected by recurrence after treatment. In fact, lesions may not be limited to a single lung segment, but the whole respiratory structure may be affected to differing degrees, which may result in persistent respiratory symptoms after surgery that tend to regress spontaneously. 

Lobectomy is more appropriate even to prevent the rare risk of malignant transformation (rhabdomyosarcoma, pulmonary blastoma), which is most frequently described in isolated or associated CCAM [21,22].

**Figure 3:**
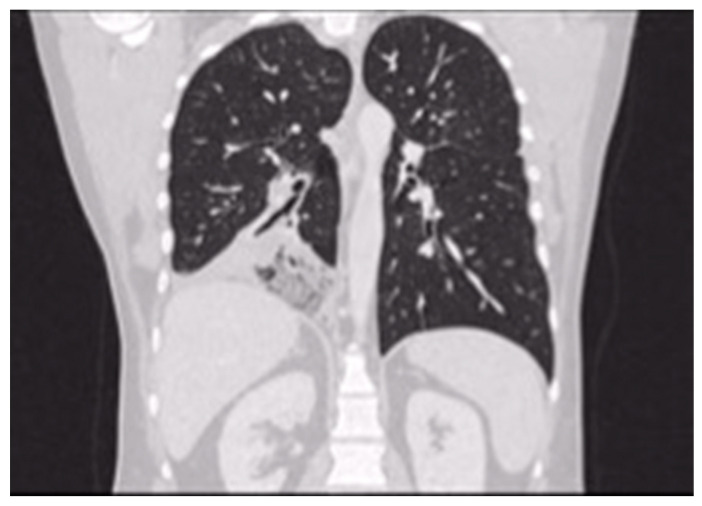
Lung cancer abscess in the residual right lower lobe in a patient treated with pulmonary segmentectomy for CCAM.

According to the literature, patients undergoing parenchyma-saving resection are likely to have early postoperative morbidities such as recurrent pulmonary infection or prolonged air leakage, and in cases of residual lesion, even malignant transformation [23]. The systematic review by Stanton et al. demonstrated a 15% rate of residual disease after segmental resection vs. 0% with lobectomy [16]. In our experience, a patient treated for CCAM by B9 segmentectomy at the age of 13 developed successive malignant transformation 14 years later (bilaterally metastatic adenocarcinoma) in the residual right lower lobe, primarily identified by respiratory symptoms occurrence (productive coughing) and radiological evidence of lung cancer abscess (Figure 3). In fact, performing subtotal lobectomy or segmentectomy or at worst, an atypical resection, would risk leaving remnants of the lesion that could later develop into lung cancer. 

Our study has several limitations: data were retrospectively collected; the study population was rather small and so the power of our results is not so strong as to be conclusive; parenchyma-saving resection was performed in only selected cases (peripheral lesions, smaller than 3 cm in diameter). However, considering the rarity of the pathology, this is one of the largest studies in literature on the topic. 

A prospective, randomized controlled trial would be helpful to determine if parenchyma-saving resection could be justified in patients with localized CPMs.

## 5. Conclusions

Surgical excision has been proven to be the treatment of choice for CPMs although a general consensus on the best type of surgery is not yet proven. To the best of our knowledge, despite the small sample size due to the rarity of the pathology described, the present multicentric study is one of the largest in literature comparing the two different surgical approaches (lobectomy vs. sub-lobar resections) for CPMs.

Despite the limits of a small sample and retrospective work, the present study aims to demonstrate the most appropriate surgical treatment for the stable remission of CPMs in both adult and paediatric patients. In conclusion, lobectomy seems to be the treatment of choice compared to sub-lobar resection, resulting in a lower or null rate of intra- and postoperative complications, a lower incidence of respiratory symptom recurrence, and preventing the rare risk of possible future malignant transformation. 

## Figures and Tables

**Table 1:** Descriptive statistical of the population.

	Lobectomy (Group A) (*n* = 50)	Segmentectomy (Group B) (*n* = 25)	*p* (<0.05)
Sex (*n*; %)			
M	30; 60%	12; 48%	
F	20; 40%	13; 52%	0.338
Symptoms (*n*; %)			
Yes	17; 34%	6; 24%	
No	33; 66%	19; 76%	0.271
Incidental diagnosis (*n*; %)			
Yes	1; 2%	6; 24%	
No	49; 98%	19; 76%	0.005
Prenatal diagnosis (*n*; %)			
Yes	42; 84%	18; 72%	
No	8; 16%	7; 28%	0.178
Histology (*n*; %)			
CCAM + SEQ			
Yes	29; 58%	8; 32%	
No	21; 42%	17; 68%	0.030
Histology CCAM (*n*; %)			
Yes	19; 38%	2; 8%	
No	31; 62%	23; 92%	0.005
Histology SEQ (*n*; %)			
Yes	2; 4%	15; 60%	
No	48; 96%	10; 40%	0.000
Side (*n*; %)			
Right	28; 56%	10; 40%	
Left	22; 44%	15; 60%	0.144
Surgical access (*n*; %)			
VATS	31; 62%	16; 64%	
Minithoracothomy	19; 38%	9; 36%	1.000
Lesions dimensions in cm (mean ± SD)	6.6 ± 4	2.5 ± 3	0.005
Intra-operatory complications (*n*; %)			
Yes	2; 4%	6; 24%	
No	48; 96%	19; 76%	0.014
Post-operatory complications (*n*; %)			
Yes	5; 10%	3; 12%	
No	45; 90%	22; 88%	0.538
Recurrence (*n*; %)			
Yes	2; 4%	6; 24%	
No	48; 96%	19; 76%	0.014
Surgery time (*n*; %)			
<120 min	18; 36%	8; 32%	
>120 min	32; 64%	17; 68%	0.469
Days of hospital stay (*n*; %)			
<7 days	38; 76%	16; 64%	
>7 days	12; 24%	9; 36%	0.205
Days of chest tube (*n*; %)			
<7 days	44; 88%	16; 64%	
>7 days	6; 12%	9; 36%	0.018
Neoplasm (*n*; %)			
Yes	2; 4%	1; 4%	
No	48; 96%	24; 96%	0.710
Age (mean ± SD)	12.70 ± 11.90	4.20 ± 11.60	0.012
Length of in-hospital stay (mean ± SD)	8.80 ± 9.11	8.40 ± 6.63	0.846
Surgery time (mean ± SD)	167.38 ± 62.38	161.48 ± 55.44	0.690
Length of chest tube permanence (mean ± SD)	3.74 ± 4.14	6.64 ± 3.94	0.005

**Table 2:** Statistical description of symptom recurrence.

	Recurrence (*n* = 8)	No Recurrence (*n* = 67)	*p* (<0.05)
Sex (*n*; %)			
M	5; 62.5%	37; 55.223%	
F	3; 37.5%	30; 44.776%	0.499
Pre-operatory symptoms (*n*; %)			
Yes	2; 25%	21; 31.343%	
No	6; 75%	46; 68.656%	0.532
Incidental diagnosis (*n*; %)			
Yes	1; 12.5%	6; 8.955%	
No	7; 87.5%	61; 91.044%	0.562
Prenatal diagnosis (*n*; %)			
Yes	7; 87.5%	53; 79.104%	
No	1; 12.5%	14; 20.895%	0.495
Histology CCAM + SEQ (*n*; %)			
Yes	2; 25%	35; 52.238%	
No	6; 75%	32; 47.761%	0.140
Histology CCAM (*n*; %)			
Yes	0; 0%	21; 31.343%	
No	8; 100%	46; 68.656%	0.062
Histology (*n*; %)			
Yes	6; 75%	11; 16.417%	
No	2; 25%	56; 83.582%	0.001
Side (*n*; %)			
Right	6; 75%	32; 47.761%	
Left	2; 25%	35; 52.238%	0.140
Intra-operatory complications (*n*; %)			
Yes	3; 37.5%	5; 7.462%	
No	5; 62.5%	62; 92.537%	0.035
Post-operatory complications (*n*; %)			
Yes	3; 37.5%	5; 7.462%	
No	5; 62.5%	62; 92.537%	0.035
Surgery time (*n*; %)			
<120 min	2; 25%	24; 35.82%	
>120 min	6; 75%	43; 64.179%	0.428
Days of hospital stay (*n*; %)			
<7 days	6; 75%	48; 71.641%	
>7 days	2; 25%	19; 28.358%	0.604
Days of chest tube (*n*; %)			
<7 days	7; 87.5%	53; 79.104%	
>7 days	1; 12.5%	14; 20.895%	0.495
Neoplasm (*n*; %)			
Yes	2; 25%	1; 1.492%	
No	6; 75%	66; 98.507%	0.029
Surgery (*n*; %)			
Lobectomy	2; 25%	48; 71.641%	
Segmentectomy	6; 75%	19; 28.358%	0.014

**Table 3:** Univariate and multivariate logistic regression.

	*p*	OR (95% CI)	*p*	OR (95% CI)
Sequestration	0.002	15.273 (2719–85.797)		
Segmentectomy	0.019	7579 (1404–40.917)	0.040	10.412 (0.958–113.096)
Intraop Compl	0.020	7440 (1364–40.595)	0.105	6629 (0.674–65.180)
Postop Compl	0.020	7440 (1364–40.595)	0.022	14.279 (1472–138.461)
Neoplasm	0.017	22.000 (1732–279.449)	0.062	33.298 (0.842–1316.492)

## Data Availability

No new data were created or analyzed in this study. Data sharing is not applicable to this article.
